# Hypertensive disorders of pregnancy and midlife maternal cognition in a prospective cohort study

**DOI:** 10.1111/jch.14765

**Published:** 2024-01-12

**Authors:** Kate Birnie, Janet Catov, Emma L. Anderson, Winok Lapidaire, Fanny Kilpi, Deborah A. Lawlor, Abigail Fraser

**Affiliations:** ^1^ Population Health Sciences, Bristol Medical School Bristol UK; ^2^ MRC Integrative Epidemiology Unit at the University of Bristol, Oakfield House, Oakfield Grove Bristol UK; ^3^ Department of Obstetrics and Gynecology University of Pittsburgh Pittsburgh Pennsylvania USA; ^4^ Magee‐Womens Research Institute Pittsburgh Pennsylvania USA; ^5^ Department of Mental Health of Older People Division of Psychiatry University College London London UK; ^6^ Division of Cardiovascular Medicine Radcliffe Department of Medicine University of Oxford Oxford UK

**Keywords:** ALSPAC, cognitive function, gestational hypertension, pre‐eclampsia

## Abstract

Hypertensive disorders of pregnancy (HDP) are associated with an increased risk of cardiovascular disorders, with recent evidence linking pre‐eclampsia with vascular dementia. We examined associations of HDP with cognitive performance measured in midlife, in a prospective cohort study, the Avon Longitudinal Study of Parents and Children. Six cognitive function domains were measured 20 years after pregnancy at a mean age of 51 years. The cognition tests were repeated at clinics in the following two years. Cognitive function domains measured were immediate and delayed verbal episodic memory, working memory, processing speed, verbal intelligence, and verbal fluency. Exposures were pre‐eclampsia, gestational hypertension (GH), and a combined category of any HDP, all compared to normotensive pregnancy. Of 3393 pregnancies included in the analysis, GH was experienced by 417 (12.3%) and pre‐eclampsia by 57 (1.7%). GH was associated with lower verbal episodic memory, in the delayed logic memory test (‐0.16 SDs; 95% CI ‐0.30, ‐0.03; *p* = .015) and there was weak evidence of an association with the immediate logic memory test (‐0.13 SDs; ‐0.27, 0.001; *p* = .058). However, we did not see steeper declines by age for women with GH and there was no evidence of associations with other cognitive domains or for pre‐eclampsia with any domains. Results were not substantially changed after controlling for midlife blood pressure. Our findings suggest that a history of GH is associated with slightly reduced episodic memory 20 years after pregnancy, but we found no evidence of a quicker age‐related decline compared to women with normotensive pregnancies.

## 1 INTRODUCTION

Hypertensive disorders of pregnancy (HDP) include gestational hypertension (GH) and pre‐eclampsia and are estimated to occur in almost 10% of pregnancies.^1^, ^2^ HDP are associated with an increased risk of developing a range of cardiovascular disorders in later life including myocardial infarction, stroke, heart failure.^1^, ^3^ Evidence is emerging that during the post‐partum period, there is identifiable progression of hypertensive‐related disease within the heart, brain, and vasculature. Recently, population‐based cohort studies have shown that pre‐eclampsia is associated with an increased risk of vascular dementia.^4^, ^5^ It is plausible that HDP could be linked with lower cognitive function in midlife, prior to impairments and diagnoses in older age.

A systematic review examining the association between a history of pre‐eclampsia and cognitive function later in life found that pre‐eclampsia was associated with subjective cognitive symptoms, but no clear evidence of impairment on standard neurocognitive tests.^6^ All studies included in the systematic review were case‐control, retrospective cohort, or cross‐sectional studies; findings of self‐reported cognitive decline in formerly pre‐eclamptic women could be susceptible to recall bias. The majority of studies reported the measure of cognitive less than 10 years after pregnancy and women were relatively young at the time of cognition assessment.^7^, ^8^, ^9^, ^10^, ^11^ While useful to assess cognition in the short‐term after HDP, it is possible that more clinically important cognitive issues might only become apparent over a longer follow‐up. Most studies examining HDP and cognition focused either on any HDP^12^ or pre‐eclampsia only^7^, ^8^, ^9^, ^10^, ^11^, ^13^ and have not examined associations with GH alone. The authors of the systematic review concluded that future studies that follow large cohorts of women with and without HDP over a long period of time, use well‐validated and uniform neurocognitive tests assessing each cognitive domain, and attempt to control for confounders and mediators, are needed.^6^ HDP are recognized as risk factors for cardiovascular diseases.^1^, ^3^ It is therefore possible that any relationship between HDP and cognition could be explained by blood pressure, a major risk factor for cardiovascular disease. Addressing gaps in the current literature is crucial for advancing our understanding of the potential cognitive impacts of HDP.

Here, we aim to assess associations of pre‐eclampsia, GH, and any HDP, all compared to normotensive pregnancies with six domains of objectively measured cognitive performance. Cognition was assessed in midlife, approximately 20 years after pregnancy, in a large prospective cohort study. We also assess if any observed associations are explained by blood pressure measured in midlife.

## 2 METHODS

### 2.1 Study population

The Avon Longitudinal Study of Parents and Children (ALSPAC) is a prospective population‐based birth cohort study. Pregnant women resident in Avon, UK, with expected dates of delivery between April 1, 1991, and December 31, 1992, were invited to take part. The initial number of pregnancies enrolled was 14 541.^14^, ^15^ Of the original 14 541 initial pregnancies, 338 were from a woman who had already enrolled with a previous pregnancy, meaning 14 203 unique mothers were initially enrolled in the study.^16^ Detailed data were collected during pregnancy and the children and families have been followed up since birth through questionnaires, clinics and linkage to routine datasets. Please note that the study website contains details of all the data that is available through a fully searchable data dictionary and variable search tool (http://www.bristol.ac.uk/alspac/researchers/our‐data/). All mothers still engaged with the study were invited to an assessment clinic in 2011−2013 where cognition was measured. Follow‐up clinics in 2013−2014 and 2014−2015 also carried out the same cognition assessments. In the current analysis, we included women enrolled in ALSPAC and excluded women with triplet/quadruplet pregnancies, women with no obstetric data, women with very limited covariate data, women without outcome data, and women who did not consent to the cognition tests being performed. The eligible cohort was 3393 women with obstetric data from pregnancy and the cognition outcome measures (available from at least one clinic time point). A flow of participants through the study is shown in Figure S1.

### 2.2 Hypertensive disorders of pregnancy

Trained research midwives abstracted data from obstetric medical records obtained from routine clinical practice, on every measurement of systolic blood pressure (SBP), diastolic blood pressure (DBP), and proteinuria.^17^ The median number (interquartile range) of blood pressure measurements in pregnancy was 14 (11−16) and 11 (10−14) for urine measurements. All women were categorized into one of three mutually exclusive categories of GH, pre‐eclampsia, or no hypertensive disorder of pregnancy. Pre‐eclampsia was defined as a SBP ≥140 mmHg or a DBP ≥90 mmHg, measured on ≥2 occasions after 20 weeks of gestation, with proteinuria, diagnosed if the protein reading on dipstick testing was ≥1 (30 mg/dL), occurring at the same time as the elevated blood pressure.^18^ The GH exposure was defined as the same pattern of elevated blood pressure but without proteinuria. We also created a combined category of any HDP by grouping GH and pre‐eclampsia.

### 2.3 Cognition in midlife

Six different cognitive tests were administered at an assessment clinic when women had a mean age of 50.3 years (standard deviation (SD): 4.4). Women were reassessed with the same cognition tests at two further follow‐up assessment clinics, each successively 1–2 years apart, with mean ages of 51.6 years (SD: 4.6) and 52.6 years (SD: 4.4) at assessment. Tests were carried out according to a standardized protocol and assess specific domains of cognitive function.^19^ The cognition tests were: (1) immediate logic memory test (measuring immediate verbal episodic memory),^20^ (2) delayed logic memory test (measuring delayed verbal episodic memory),^20^ (3) backward digit span test (measuring working memory),^21^, ^22^ (4) digit symbol coding test (measuring processing speed),^22^ (5) spot‐the‐word test (measuring verbal intelligence),^23^ and (6) same letter word test (measuring verbal fluency).^21^ Higher scores on each test reflect better cognitive function.

We chose the first cognition assessment for each woman as our primary outcomes to avoid practice effects and maximise statistical power in parsimonious regression models; if a woman missed the first clinic, we used their assessments from a follow‐up clinic. Our secondary outcomes were the repeated cognitive function test scores across all the clinics. Cognition test scores were converted into *z*‐scores, calculated as the standardized residuals from linear regression models. These regression models controlled for the individual fieldworkers who carried out the test, to reduce any potential variation in performance related to how the tests were administered. The standardized scores have a mean of zero and a standard deviation (SD) of one, enabling the comparison for an equivalent one SD change in each cognition measurement. Standardized scores were recalculated for the secondary outcomes, accounting for fieldworker *and* the number of previous testing occasions, to control for practice effects.

### 2.4 Other variables

Information on the following covariates were obtained from questions during pregnancy: maternal age (years), household occupational social class (I, II, III non manual, III manual, IV/V), whether they smoked during pregnancy (never, temporarily, throughout pregnancy), maternal education (O level/lower, A level or higher degree/higher), parity (0, 1, 2, 3+), ethnicity (white/non‐white), pre‐pregnancy body mass index (BMI; < 18.5, 18.5‐24.9, 25−29.9, 30+ kg/m^2^), whether they had a phone in their home, marital status (yes, never, other), housing tenure (own/mortgage, private rental, other), whether the mother or her partner had use of a car, whether their house had double glazing (i.e., double‐pane windows; for all, some, or none of the windows), crowding index (defined as number of people per room excluding bathrooms and toilets), whether there were any major financial difficulties since becoming pregnant. Information on depression during pregnancy (Edinburgh Postnatal Depression Scale at 18 weeks of gestation, in categories) and depression since birth (recorded at 8 months after birth; yes/no) and breast‐feeding duration (never, < 3 months, 3−5 months, or 6 months+) was taken from questionnaires administered after birth. Seated blood pressure was measured at the same clinic as the cognition tests. Two readings of SBP and DBP were recorded, and the mean is used in analyses. If women had previously reported that they were taking medication for blood pressure, we added 10 mmHg to the recorded measure. Menopausal status (yes/no) was also ascertained during the clinic visit.

### 2.5 Statistical methods

In our primary analysis, linear regression models were used to investigate associations of (1) GH, (2) pre‐eclampsia, and (3) any HDP compared with normotensive pregnancy, for the six cognition standardized test scores from the first assessment. We focus our interpretation of the results on the size of associations and their confidence intervals (CIs), rather than arbitrary *p* value thresholds.^24^ No adjustments to *p* values were made for multiple testing. We considered education and demographic factors as possible confounding factors that could influence the risk of HDP and also impact on the test results in the cognitive outcomes. Models controlled for the following potential confounders: age at the cognition test, age at birth of index child, household social class, smoking status, parity, ethnicity, and body mass index. We used robust standard errors (SEs) to account for non‐independence as a small number of women appeared in the cohort for more than one pregnancy. To address the potential selection bias due to women dropping out of the longitudinal study over time, the regression models had inverse probability weights (IPW) applied. Weights were generated based on predictors of continued participation with the cohort. The model for weights included predictors of maternal participation^25^: education, parity, age at first pregnancy and birth of index child, ethnicity, family social class, smoking, duration of breastfeeding, marital status, housing tenure, whether there was a phone in the home, car use, double glazing, financial difficulties, crowding index, and depression. Each woman who remained in the study was given a weight of the inverse of their probability of staying in the study; this method gives more weight to individuals who have similar characteristics to those who dropped out of the study. Multiple imputation using chained equations^26^ was used to impute missing data in the covariates. The imputation model included all covariates as well as outcomes and exposures. IPWs were calculated in each of 25 imputed datasets, and weighted estimates for each standardized outcome were pooled across the imputed datasets. To assess whether variations in blood pressure could account for/attenuate any associations of HDP and midlife maternal cognition, we ran models that additionally controlled for blood pressure measured at the same clinics as the cognition assessment. There may be changes in cognitive function in women going through the menopause; to assess whether any associations were changed after accounting for the menopause, we included a variable on menopausal status ascertained at the clinic. Weighted analyses can be sensitive to outlying weights that can unduly influence results or give large CIs. We carried out a sensitivity analysis truncating the IPW at the 99^th^ and 95^th^ percentiles to see if associations were being influenced by large IP weights.

In the analysis of the secondary outcomes, we used multilevel linear regression models to examine change in the standardized cognitive function domains by age, using data from all three cognitive assessments. Multilevel models allow all women with at least one cognitive function assessment to be included in analyses under a missing‐at‐random assumption. We adjusted for potential confounders/covariates (household social class, maternal smoking in pregnancy, maternal education, pre‐pregnancy BMI, ethnicity) and applied IPW to control for the effects of selection due to attrition from the study. Models included random intercepts per woman to allow for individual variation in cognitive function at baseline. To explore whether a decline in cognitive function with age differed by HDP status, we fitted with an interaction term between the GH and pre‐eclampsia exposures and age. We plotted the predicted trajectory of cognitive performance by age, with 95% CI. The predictions were made for the following categories of the covariates: household occupational social class II, never smoked during pregnancy, maternal education of O level/lower, nulliparous, white ethnicity, BMI 18.5–24.9 kg/m^2^.

## 3 RESULTS

### 3.1 Characteristics of the study population

The characteristics of the 3393 women included in the analysis with outcome data are shown in Table S1, which compares them to women who were in the original cohort but not included in this analysis, before multiple imputation of missing data for the covariates. Women included in the analysis were older at their first pregnancy compared to without follow‐up data 20 years after pregnancy, and were therefore not included in the analysis (mean age 26.3 vs. 23.7 years). Included women were more likely to be from household social class I or II compared to women who were not included (64.0% vs. 41.1%). Of the women included in the analysis, 20.9% had a degree or higher listed as their educational attainment, compared to 8.7% women who were not included in the analysis. Women included in the analysis had a lower depression score in pregnancy (30.1% had a score of 0−3 compared to 22.7% women not included in the analysis) and were more likely to report they were not depressed at 8 months after birth (70.2% compared to 52.3% in women who were not included in the analysis). Table S2 shows the mean (SD) of the cognition test scores prior to standardisation. Pre‐eclampsia was experienced by 57 women (1.7%) and GH was experienced by 417 women (12.3%), 474 women (14.0%) experienced any HDP. Table 1 shows the baseline characteristics of the included women by HDP status. Women who experienced HDP were more likely to have a parity of 0 (61.6% compared to 43.9% in women with normotensive pregnancies) and have a BMI of 25 or higher (26.2% compared to 12.6% women with normotensive pregnancies).

**TABLE 1:** Baseline characteristics of study participants by HDP status, mean (SD), or number (%).

		Normotensive pregnancy	HDP
Age at index pregnancy	Years	30.0 (4.4)	29.6 (4.8)
Family social class	I	548 (18.8%)	83 (17.5%)
	II	1325 (45.4%)	215 (45.4%)
	III Non manual	631 (21.6%)	106 (22.4%)
	III Manual	214 (7.3%)	28 (5.9%)
	IV/V	69 (2.4%)	12 (2.5%)
	Missing	132 (4.5%)	30 (6.3%)
Maternal education	O level/lower	1398 (47.9%)	231 (48.7%)
	A level	862 (29.5%)	134 (28.3%)
	Degree/higher	614 (21.0%)	94 (19.8%)
	Missing	45 (1.5%)	15 (3.2%)
Parity	0	1281 (43.9%)	292 (61.6%)
	1	1073 (36.8%)	114 (24.1%)
	2	387 (13.3%)	47 (9.9%)
	3+	123 (4.2%)	15 (3.2%)
	Missing/uncertain	55 (1.9%)	6 (1.3%)
Ethnicity	White	70 (2.4%)	6 (1.3%)
	Non white	2799 (95.9%)	451 (95.1%)
	Missing	50 (1.7%)	17 (3.6%)
Pre pregnancy BMI	<18.5	138 (4.7%)	10 (2.1%)
	18.5 ‐ 24.9	2217 (76.0%)	309 (65.2%)
	25−29.9	296 (10.1%)	97 (20.5%)
	30+	74 (2.5%)	27 (5.7%)
	Missing	194 (6.6%)	31 (6.5%)

### 3.2 Main analyses

#### 3.2.1 Associations between HDP with cognitive performance in midlife

**FIGURE 1:**
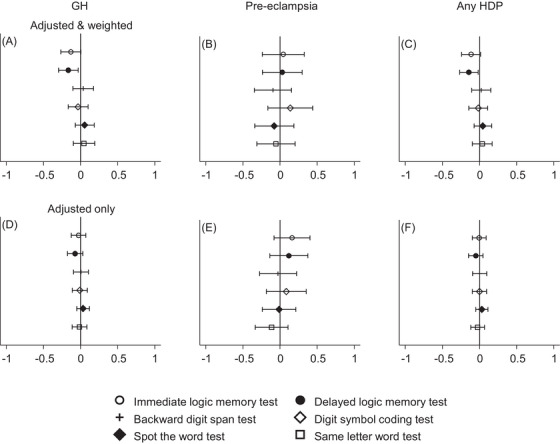
Difference in standardized cognitive test scores by hypertensive disorders of pregnancy. The x axis shows the standard deviation difference for each test score; higher scores reflect better cognitive function. The reference category in each analysis is no hypertensive disorder of pregnancy. “Adjusted and weighted” models control for age at the cognition test, age at birth of index child, household social class, smoking status, parity, ethnicity, and body mass index and apply IPW to control for the effects of selection due to attrition from the study (see Methods section for further details). “Adjusted only” models control for the variables mentioned above, but do not apply IPW.

In models for the primary outcome (the first cognition assessment), having experienced GH was associated with lower scores in the delayed logic memory test (regression coefficient −0.16 SDs; 95% CI −0.30, −0.03; *p* = .015), which measure verbal episodic memory (Figure 1A, Table 2). There was also weak evidence for an association between GH and lower scores in the immediate logic memory test (−0.13 SDs; −0.27, 0.001; *p* = .058). These models control for baseline covariates and apply IPW to control for selection bias. There was no evidence of associations with the backward digit span test, spot the word test, same letter word test, or for the pre‐eclampsia exposure (Figure 1A,B, Table 2). There was no evidence of associations between HDP exposures and measures of verbal episodic memory in the models that did not include IPW (the “adjusted only” results are shown in Figure 1D‐F). These results suggest there could be evidence of selection bias in non‐weighted analyses. Results from unadjusted, but weighted models, are shown in Figure S2 for completeness.

**TABLE 2:** Difference in standardized cognitive test scores (95% confidence interval): Results from multivariable adjusted and weighted regression models, by hypertensive disorders of pregnancy (HDP).

	Immediate logic memory test	Delayed logic memory test	Backward digit span test	Digit symbol coding test	Spot the word test	Same letter word test
Adjusted and weighted models						
GH	−0.13 (−0.27, 0.001)	−0.16 (−0.30, −0.03)	0.04 (−0.10, 0.17)	−0.03 (−0.17, 0.10)	0.06 (−0.07, 0.18)	0.05 (−0.10, 0.18)
*p*	.058	.015	.618	.627	.393	.528
Pre‐eclampsia	0.04 (−0.24, 0.33)	0.03 (−0.24, 0.30)	−0.10 (−0.35, 0.16)	0.14 (−0.17, 0.44)	−0.08 (−0.34, 0.18)	−0.06 (−0.31, 0.20)
*p*	.763	.827	.450	.376	.558	.670
Any HDP	−0.11 (−0.24, 0.01)	−0.15 (−0.27, −0.02)	0.02 (−0.11, 0.15)	−0.02 (−0.15, 0.11)	0.04 (−0.08, 0.16)	0.04 (−0.10, 0.17)
*p*	.081	.022	.748	.773	.476	.605
Additionally controlling for blood pressure						
GH	−0.13 (−0.27, 0.01)	−0.18 (−0.32, −0.05)	0.06 (−0.08, 0.20)	−0.01 (−0.15, 0.13)	0.06 (−0.07, 0.19)	0.04 (−0.11, 0.19)
*p*	.060	.008	.402	.861	.364	.612
Pre‐eclampsia	0.04 (−0.24, 0.33)	0.01 (−0.26, 0.28)	−0.07 (−0.32, 0.18)	0.17 (−0.13, 0.47)	−0.06 (−0.33, 0.21)	−0.06 (−0.32, 0.20)
*p*	.764	.962	.585	.274	.642	.661
Any HDP	−0.11 (−0.24, 0.02)	−0.16 (−0.29, −0.04)	0.05 (−0.09, 0.18)	0.01 (−0.13, 0.13)	0.05 (−0.07, 0.17)	0.03 (−0.11, 0.17)
*p*	.085	.012	.491	.977	.434	.677

*Note*: “Adjusted and weighted models” control for age at the cognition test, age at birth of index child, household social class, smoking status, parity, ethnicity, and body mass index, and apply IPW to control for the effects of selection due to attrition from the study (see Methods Section for further details).

Abbreviations: GH, gestational hypertension; HDP, hypertensive disorders of pregnancy.

#### 3.2.2 Controlling for midlife blood pressure and menopausal status

We controlled for blood pressure measured in midlife to see if this explained the observed associations of GH with cognitive test scores, but results were not substantially changed (Table 2, Figure S3). Further investigation showed that blood pressure in mid‐life was not strongly or consistently related to the cognition outcomes (Figures S4 and S5) but women who experienced GH and pre‐eclampsia in pregnancy had higher blood pressure in midlife (Figure S6). Results were not changed when accounting for menopausal status (Figure S7). The median value of IPW was 1.4 (range 1.0–54.0).

### 3.3 Sensitivity analyses

In sensitivity analyses we truncated the IPW at the 99^th^ percentile (a weight of 10.9) and at the 95^th^ percentile (a weight of 4.7). Compared to the fully adjusted and weighted model, the coefficients for the delayed and immediate logic memory tests were similar but attenuated when truncating the IPW and CIs also narrowed (Figure S8). For example, the coefficients for GH and the delayed logic memory tests after truncating the IPW at the 99^th^ and 95^th^ percentiles were −0.14 SDs (95% CI −0.26, −0.02; *p* = .023) and −0.10 SDs (95% CI −0.21, 0.01; *p* = .086), respectively.

### 3.4 Secondary analyses

#### 3.4.1 Age and cognitive performance

**FIGURE 2:**
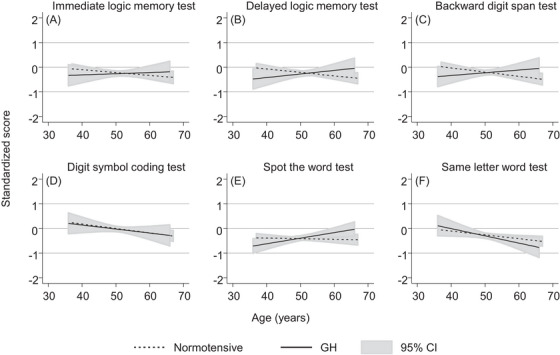
The average cognition trajectories by gestational hypertension (GH) status, with 95% confidence intervals. The standardized scores control for fieldworker and number of previous testing occasions. Models control for age at the cognition test, age at birth of index child, household social class, smoking status, parity, ethnicity, and body mass index and apply IPW to control for the effects of selection due to attrition from the study (see Methods section for further details). The y axis shows the standard deviation difference for each test score; higher scores reflect better cognitive function. “Normotensive” represents no hypertensive disorder of pregnancy.

**FIGURE 3:**
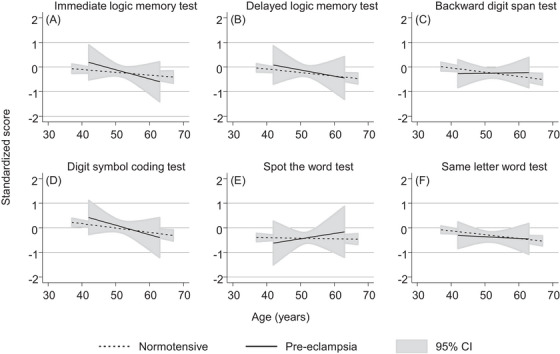
The average cognition trajectories by pre‐eclampsia status, with 95% confidence intervals. The standardized scores control for fieldworker and number of previous testing occasions. Models control for age at the cognition test, age at birth of index child, household social class, smoking status, parity, ethnicity, and body mass index and apply IPW to control for the effects of selection due to attrition from the study (see Methods section for further details). The y axis shows the standard deviation difference for each test score; higher scores reflect better cognitive function. “Normotensive” represents no hypertensive disorder of pregnancy.

The results from the analysis of the secondary outcomes, using the repeated cognitive function test scores across the repeated clinics are shown in Figures 2 and 3. We observed that cognitive performance declined with age, across all cognitive function domains, for women with normotensive pregnancies. The cognitive outcome with the steepest predicted decline by age was for the digit symbol coding test. For all outcomes, the CIs around the predicted trajectories were wide for women who experienced GH and pre‐eclampsia and the CIs overlapped with the predicted trajectories for women who were normotensive. The predicted trajectory for women with GH for the delayed logic memory test was lower than the predicted trajectory for women with normotensive pregnancies for the younger ages (e.g., aged 40 years), however, a steeper decline with age was not observed. Similar patterns were observed for GH for the immediate logic memory test and backward digit span test. Predicted trajectories for women who experienced pre‐eclampsia were broadly similar to predicted trajectories for women who experienced normotensive pregnancies, with wide CIs.

## 4 DISCUSSION

### 4.1 Summary of findings

This prospective, longitudinal cohort study revealed some evidence that women who experienced GH had lower cognitive performance 20 years post‐partum compared to women who experienced a normotensive pregnancy, in tests that measure verbal episodic memory. These associations were observed in linear regression models that adjusted for confounding and used weighting to take account of attrition from the cohort study, but evidence of associations was not observed for these outcomes for women who experienced pre‐eclampsia. Episodic memory was measured using the immediate and delayed logic memory tests, which capture ability to remember experienced events and other familiar, contextual information shortly after the events. In the immediate version of the test, the women were told a “story” in a standardized way (via a tape recording) then asked about key facts immediately after the “story” was completed.^22^ In the delayed version of the tests, the women were asked to recall key facts after undergoing other cognitive tests (∼10 min after hearing the story).

### 4.2 Explanation of findings and comparison with previous studies

We observe age‐related declines in all domains of cognitive performance, including episodic memory, in women who had not experienced any HDP, with these findings consistent with age‐related decline in cognitive function, as shown previously in the same cohort^19^ and elsewhere.^27^, ^28^ However, rates of age‐related change across all of these domains were similar in women who had and who had not experienced HDP. This suggests that the small differences in episodic memory in women who have experienced GH 20 years after pregnancy is not due to a hastening of age‐related decline and may be something that was present earlier, for example, immediately postnatal. ALSPAC does not have measures of cognitive function until ∼20 years after the index pregnancy to explore this. Observed differences in standardized cognitive test scores comparing women with GH and women with normotensive pregnancies for the episodic memory outcome were not large, regression coefficient: −0.16 SDs (95% CI −0.30, −0.03) for the delayed and −0.13 SDs (−0.27, 0.001) for the immediate logic memory tests. Putting our results into context, an intervention of aerobic exercise on improving episodic memory in late adulthood observed a standardized mean difference of 0.28; (95% CI 0.10, 0.46).^29^ Furthermore, a meta‐analysis of population‐based cohorts observed an association of current versus never smoking of −0.14 SDs (regression coefficient for a global cognition score, SE 0.17) at an average age of 72.7 years.^30^ Our subtle differences in episodic memory in women with GH might translate into real‐world consequences for affected individuals. For example, if reduced episodic memory capabilities impact on daily activities and quality of life. It would be useful for large prospective studies to evaluate cognitive outcomes after hypertensive disorders of pregnancy, with a greater number of repeated measurements over a wider age span to determine whether a reduction in episodic memory ability is replicated and persists into older age.

For the verbal episodic memory outcome, we observed associations for GH and the combined exposure of any HDP, but not for the exposure of pre‐eclampsia alone. Results from the sensitivity analysis truncating the weights suggested that findings for GH were not due to excessive outlying IPWs, rather we observed an improvement to the variance at the cost of potentially reintroducing a small amount of selection bias. Our observed associations for GH could be due to chance. Or our lack of observed association for the pre‐eclampsia group could be due to lower numbers with this condition. If pre‐eclampsia is a more severe version of GH, we would expect to also see similar associations for pre‐eclampsia. However, there is still uncertainty about whether GH and pre‐eclampsia are the same disease, but differ in severity, or whether they are distinct conditions.^31^, ^32^, ^33^ Pre‐eclampsia is not solely a blood pressure condition and involves other systems such as inadequate placentation.^34^ The observed association for GH was not attenuated when controlling for midlife blood pressure, suggesting there could be other mechanisms that explain associations, but what these mechanisms could be remains unclear because the pathophysiology and sequalae of HDP are not completely understood. We did not see a steeper decline in cognitive performance for women with GH for the episodic memory outcomes. It might be that the observed difference in cognitive performance in women with GH in early midlife compared to normotensive women emerged prior to pregnancy or post‐partum. Unfortunately, cognition was not assessed at earlier time points, and we are therefore unable to test this. Moreover, when we examine change over time, the initial difference observed at age 50 does not persist, though CIs are wide. Determining whether a differential decline across exposure categories emerges later in the life would require further follow up in ALSPAC and/or additional studies with cognitive assessment in later ages.

A longitudinal prospective observational study of 30 women at the University of Pittsburgh Medical Center Magee Women's Hospital, Pittsburgh, USA, found that women with a diagnosis of pre‐eclampsia reported worse subjective memory and lower scores in attention, working memory, and executive function domains during the third trimester of pregnancy and shortly after pregnancy (up to three months postpartum).^35^ A systematic review examining association between a history of pre‐eclampsia and cognitive function later in life found that pre‐eclampsia was associated with subjective cognitive symptoms, but there was no clear evidence of impairment on standard neurocognitive tests^6^ and all studies included in the systematic review were case‐control, retrospective cohort, or cross‐sectional studies. Median time since pregnancy was only 6 years and there was marked between‐study heterogeneity, which prevented pooling results across studies for most cognitive outcomes. Maternal vascular malperfusion (MVM) is a novel placental risk marker that can give insight into vascular impairments after delivery. A study of 45 women observed reduced information processing speed 10 years after delivery among women with pre‐eclampsia and MVM compared to women without these conditions, suggesting a vascular contribution to cognitive impairment later in life.^36^ The study included women who delivered a liveborn, singleton infant at Magee‐Women's Hospital and who had a clinical placenta pathology report. The authors noted that their study sample included a large proportion (42.2%) of Black women. Prior work has primarily been in white women, yet hypertensive disorders of pregnancy and pregnancy‐related morbidity and mortality are disproportionately experienced by Black women. A recent nested cohort study embedded within the Generation R Study, an ongoing population based prospective birth cohort in Rotterdam, found that women with a history of HDP had reduced working memory and verbal learning 15 years after pregnancy.^37^ Cognitive function was assessed with cognitive tests in 115 women with a history of HDP and in 481 women with normotensive pregnancy. Regression coefficients for standardized test scores were of a similar magnitude to what we observed in this study, for example, women with HDP had a 0.25 SD reduction in the immediate recall test compared to women with a previous normotensive pregnancy (regression coefficient −0.25 SDs, 95% CI −0.44 to −0.06). Study authors noted that associations were mainly driven by women with GH, not pre‐eclampsia. In the US CARDIA study, cognition was measured using validated neurocognitive tests approximately 18 years after delivery. They included 568 parous women (193 with pre‐eclampsia and 375 controls with normotensive pregnancy) without baseline neurological disease or depression; the controls were matched according to delivery period. They found that pre‐eclamptic women scored subtly lower on cognitive tests related to psychomotor speed and executive function but not working memory, compared to women who experienced normotensive pregnancy.^38^ Associations were attenuated after controlling for mediating variables of blood pressure, BMI and psychosocial factors, which were reported at the time of outcome measurement. Authors speculate that their observed associations were driven by metabolic and psychosocial factors rather than an independent effect of pre‐eclampsia, and that pre‐eclampsia may be a marker of poor neurovascular health, however pre‐eclampsia was self‐reported in this cohort and susceptible to recall misclassification. The Mayo Clinic Study of Aging examined associations between HDP and type with global and domain‐specific cognitive decline in 2239 older women with a median age of 73 years.^39^ They found no difference in age‐ and education‐ adjusted cognitive performance at baseline by HDP status, but women with a history of preeclampsia had faster declines in global cognition and attention/executive function over 5 years of follow‐up. Adjusting for cardiovascular risk factors and conditions did not attenuate any of the results.

### 4.3 Strengths and limitations

Strengths of our study include the prospective design, the duration of follow‐up, and the availability of data on a range of objective cognition outcomes and potential confounders. In addition, the availability of repeat measures of maternal blood pressure and proteinuria in pregnancy allowed us to apply a standard international definition of HDP to all women and not rely on clinical diagnoses for these outcomes. However, although the ALSPAC cohort is reasonably large, the number of women with HDP, and pre‐eclampsia in particular, is limited; predicted trajectories of cognitive performance by age had wide CIs. We used definitions for HDP that applied to the early 1990s when study pregnancies occurred and it is important to note that diagnosis of HDP has changed over the past 3 decades. The secondary outcomes used data from all three cognitive assessments. However, meaningful cognitive changes may take longer than the two‐year interval between measurements to develop. Furthermore, the closely spaced testing occasions created practice effects which may not have been adequately accounted for in the creation of standardized scores. The testing occasions cover a narrow age range, with no cognitive measures between the immediate postnatal period and 20 years later and no measures after the women were in their early 50s. Future work with more measures over a longer period are needed to understand whether HDP might influence cognitive performance in the early postnatal period and later in life. Another limitation is the loss of participants from the study over time. We attempted to address any potential bias due to attrition by applying inverse probability weights. The analysis which includes midlife blood pressures in the models assumes there is no intermediate variable‐outcome confounding and this assumption may not be met, although we have controlled for pre‐pregnancy BMI in the adjusted and weighted models. While the test for verbal intelligence has been shown to be valid and reliable,^23^ the tests for verbal episodic memory and working memory could be at risk of misclassifying mild cognitive impairment in healthy older adults.^40^ However, we controlled for education and demographic variables in regression models to reducing the likelihood of misclassifying lower cognitive ability. Finally, we only have information on a single pregnancy, not every pregnancy for the women included in the study. We might be misclassifying the exposure which could attenuate results.

## 5 CONCLUSIONS

Our findings suggest that a history of GH is associated with slightly reduced episodic memory 20 years after pregnancy, but we found no evidence of a quicker age‐related decline compared to women with normotensive pregnancies.

## AUTHOR CONTRIBUTIONS

Abigail Fraser, Deborah A. Lawlor, and Janet Catov designed the study. Kate Birnie carried out the analyses. All authors contributed to interpreting results. Kate Birnie wrote the initial paper draft with all other authors editing and providing feedback on drafts.

## CONFLICT OF INTEREST STATEMENT

Deborah A. Lawlor has received funding from Medtronic Ltd and Roche Diagnostics for research unrelated to this project.

## Supporting information

Supporting information

## Data Availability

Access to ALSPAC data is through a system of managed open access (http://www.bristol.ac.uk/alspac/researchers/access/).
