# Correction to “Hypertensive Disorders of Pregnancy and Midlife Maternal Cognition in a Prospective Cohort Study”

**DOI:** 10.1111/jch.70273

**Published:** 2026-05-06

K. Birnie, J. Catov, E.L. Anderson, et al. “Hypertensive Disorders of Pregnancy and Midlife Maternal Cognition in a Prospective Cohort Study.”  *Journal of Clinical Hypertension* 26, no. 2 (2024): 166–176, https://doi.org/10.1111/jch.14765.

Errors were identified in two of the grant codes listed in the published article. BHF grant code AA/18/1/34219 was incorrectly displayed as “AA/18/7/34219” (with a 7 in the middle‐right segment instead of a 1) and Wellcome Trust grant code 092830/Z/10/Z was incorrectly displayed with an added prefix “WT092830/Z/10/Z”.

Therefore, the following text in the Acknowledgments:

This research was specifically funded by the British Heart Foundation (Grant Ref: SP/07/008/24066, PG/19/21/34190, CH/F/20/90003 and AA/18/7/34219), Wellcome Trust (Grant Ref: WT092830/Z/10/Z), and Lifelong Health and Wellbeing (LLHW) via the MRC (Grant reference G1001357).

 should have read:

This research was specifically funded by the British Heart Foundation (Grant Ref: SP/07/008/24066, PG/19/21/34190, CH/F/20/90003 and AA/18/1/34219), Wellcome Trust (Grant Ref: 092830/Z/10/Z), and Lifelong Health and Wellbeing (LLHW) via the MRC (Grant reference G1001357).

We apologize for this error.

