# Supplementary material for: Hypertensive disorders of pregnancy and midlife maternal cognition in a prospective cohort study
Source: J Clin Hypertens (Greenwich). 2024 Jan 12;26(2):166–76. doi: 10.1111/jch.14765 (PMC10857467; doi:10.1111/jch.14765)
Supplement: Supplementary file 1 — Supporting information [file JCH-26-166-s001.docx]

**Hypertensive disorders of pregnancy and midlife maternal cognition in a prospective cohort study: Supplement**

**Figure S1 Flow of participants through the study**

IPW: inverse probability weights. ^1^ Limited covariate data was defined as when 11 or more of the 16 variables used in the calculation of IPW were missing.

**Table S1 Comparison of women who were not included in the analysis to women who were included in the analysis, mean (SD) or number (%)**

| **Covariate** | **Unit or category** | **Not in analysis**  **(N= 9,860)** | **Included in analysis**  **(3,393)** |
| --- | --- | --- | --- |
| Age at first pregnancy | Years | 23.7 (4.9) | 26.3 (4.8) |
| Age at index pregnancy | Years | 27.3 (4.9) | 29.9 (4.4) |
| Family social class | I | 877 (8.9) | 631 (18.6) |
|  | II | 3170 (32.2) | 1540 (45.4) |
|  | III Non manual | 2147 (21.8) | 737 (21.7) |
|  | III Manual | 1268 (12.9) | 242 (7.1) |
|  | IV/V | 583 (5.9) | 81 (2.4) |
|  | Missing | 1815 (18.4) | 162 (4.8) |
| Maternal smoking | Never | 6628 (67.2) | 2912 (85.8) |
|  | Temporary | 759 (7.7) | 139 (4.1) |
|  | Throughout pregnancy | 2085 (21.2) | 318 (9.4) |
|  | Missing | 388 (3.9) | 24 (0.7) |
| Parity | 0 | 4107 (41.7) | 1573 (46.4) |
|  | 1 | 3239 (32.9) | 1187 (35.0) |
|  | 2 | 1379 (14.0) | 434 (12.8) |
|  | 3+ | 608 (6.2) | 138 (4.1) |
|  | Missing/uncertain | 527 (5.3) | 61 (1.8) |
| Ethnicity | White | 229 (2.3) | 76 (2.2) |
|  | Non white | 8463 (85.8) | 3250 (95.8) |
|  | Missing | 1168 (11.9) | 67 (2.0) |
| Phone in home | No | 1360 (13.8) | 112 (3.3) |
|  | Yes | 8122 (82.4) | 3228 (95.1) |
|  | Missing | 378 (3.8) | 53 (1.6) |
| Maternal education | O level/lower | 6178 (62.7) | 1629 (48.0) |
|  | A level | 1723 (17.5) | 996 (29.4) |
|  | Degree/higher | 862 (8.7) | 708 (20.9) |
|  | Missing | 1097 (11.1) | 60 (1.8) |
| Married | Never | 2076 (21.1) | 370 (10.9) |
|  | Yes | 6171 (62.6) | 2620 (77.2) |
|  | Other | 1221 (12.4) | 370 (10.9) |
|  | Missing | 392 (4.0) | 33 (1.0) |
| Housing tenure | Own/mortgage | 6425 (65.2) | 2941 (86.7) |
|  | Private rental | 774 (7.9) | 146 (4.3) |
|  | Other | 2238 (22.7) | 249 (7.3) |
|  | Missing | 423 (4.3) | 57 (1.7) |
| Use of car | No | 1260 (12.8) | 122 (3.6) |
|  | Yes | 8191 (83.1) | 3212 (94.7) |
|  | Missing | 409 (4.2) | 59 (1.7) |
| Double glazing in the home | All windows | 2481 (25.2) | 860 (25.4) |
|  | Some | 1743 (17.7) | 867 (25.6) |
|  | None | 4835 (49.0) | 1446 (42.6) |
|  | Missing | 801 (8.1) | 220 (6.5) |
| Crowding index | ≤0.5 | 3441 (34.9) | 1785 (52.6) |
|  | >0.5 - 0.75 | 2932 (29.7) | 1011 (29.8) |
|  | >0.75 - 1 | 2112 (21.4) | 416 (12.3) |
|  | > 1 | 765 (7.8) | 100 (3.0) |
|  | Missing | 610 (6.2) | 81 (2.4) |
| Financial difficulties | None | 7282 (73.9) | 2809 (82.8) |
|  | No effect | 130 (1.3) | 25 (0.7) |
|  | Mild effect | 384 (3.9) | 110 (3.2) |
|  | Fair effect | 333 (3.4) | 104 (3.1) |
|  | Large effect | 415 (4.2) | 92 (2.7) |
|  | Missing | 1316 (13.4) | 253 (7.5) |
| Depression score | 0-3 (least depressed) | 2239 (22.7) | 1020 (30.1) |
| (in pregnancy) | 4-6 | 1982 (20.1) | 825 (24.3) |
|  | 7-10 | 2258 (22.9) | 751 (22.1) |
|  | 11+ (most depressed) | 2104 (21.3) | 567 (16.7) |
|  | Missing | 1277 (13.0) | 230 (6.8) |
| Depression (8m after birth) | No | 5156 (52.3) | 2381 (70.2) |
|  | Yes | 2546 (25.8) | 874 (25.8) |
|  | Missing | 2158 (21.9) | 138 (4.1) |
| Breast feeding | Never | 1967 (20.0) | 411 (12.1) |
| duration | <3 months | 1568 (15.9) | 628 (18.5) |
|  | 3-5 months | 1103 (11.2) | 553 (16.3) |
|  | 6 months + | 2004 (20.3) | 1524 (44.9) |
|  | Missing | 3218 (32.6) | 277 (8.2) |

Note: Table 1 shows data before multiple imputation of missing data. Covariates in this table were used for inverse probability weighting.

**Table S2 Outcome and exposure measurements**

| **Variable** | **Variable** | **Mean (SD) or N (%)** |
| --- | --- | --- |
| **Outcomes (prior to standardisation)** | Immediate logic memory test | 15.4 (3.6) |
|  | Delayed logic memory test | 14.2 (3.8) |
|  | Backward digit span test | 7.0 (2.3) |
|  | Digit symbol coding test | 80.0 (14.2) |
|  | Spot the word test | 43.6 (7.6) |
|  | Same letter word test | 42.7 (12.3) |
| **Exposures** | GH | 417 (12.3) |
|  | Preeclampsia | 57 (1.7) |
|  | Any HDP | 474 (14.0) |

GH: Gestational hypertension; HDP Hypertensive disorders of pregnancy.

**Figure S2 Results from unadjusted and weighted regression models: difference in standardized cognitive test scores by hypertensive disorders of pregnancy**

Notes: IPW is used to control for the effects of selection due to attrition from the study. The x axis shows the standard deviation difference for each test score; higher scores reflect better cognitive function. The reference category in each analysis is no hypertensive disorder of pregnancy.

**Figure S3 Results from adjusted and weighted regression models additionally controlling for blood pressure measured in mid-life: difference in standardized cognitive test scores by hypertensive disorders of pregnancy**

Notes: Models control for baseline covariates and midlife blood pressure; IPW is used to control for the effects of selection due to attrition from the study. The x axis shows the standard deviation difference for each test score; higher scores reflect better cognitive function. The reference category in each analysis is no hypertensive disorder of pregnancy.

**Figure S4 Standardized cognition in mid-life by quintiles systolic blood pressure in mid-life**

Notes: quintiles of blood pressure go from 1 (lowest) to 5 (highest) on the x axis, and higher standardized test scores reflect better cognitive function on the y axis. These analyses are unadjusted.

**Figure S5 Standardized cognition in mid-life by quintiles of diastolic blood pressure in mid-life**

Notes: quintiles of blood pressure go from 1 (lowest) to 5 (highest) on the x axis, and higher test scores reflect better cognitive function on the y axis. These analyses are unadjusted.

**Figure S6 Blood pressures in mid-life by hypertensive disorders of pregnancy**

**Figure S7 Results from adjusted and weighted regression models additionally controlling for menopause: difference in standardized cognitive test scores by hypertensive disorders of pregnancy**

Notes: Models control for baseline covariates and menopause status; IPW is used to control for the effects of selection due to attrition from the study. The x axis shows the standard deviation difference for each test score; higher scores reflect better cognitive function. The reference category in each analysis is no hypertensive disorder of pregnancy.

**Figure S8 Difference in standardized cognitive test scores by hypertensive disorders of pregnancy, for different levels of truncating the inverse probability weights**

Notes: The x axis shows the standard deviation difference for each test score; higher scores reflect better cognitive function. The reference category in each analysis is no hypertensive disorder of pregnancy.

Multivariable models adjusting for baseline covariates and weighted using IPW, for the exposures of GH, pre-eclampsia and HDP. Models are shown using: the full weights, the weights truncated at the 99^th^ percentile and the weights truncated at the 95^th^ percentile.
